# Supplementary material for: Insulating state in tetralayers reveals an even–odd interaction effect in multilayer graphene
Source: Nat Commun. 2015 Mar 3;6:6419. doi: 10.1038/ncomms7419 (PMC4366515; doi:10.1038/ncomms7419)
Supplement: Supplementary Information — Supplementary Figures 1-2, Supplementary Notes 1-3 and Supplementary References [file ncomms7419-s1.pdf]

Supplementary information

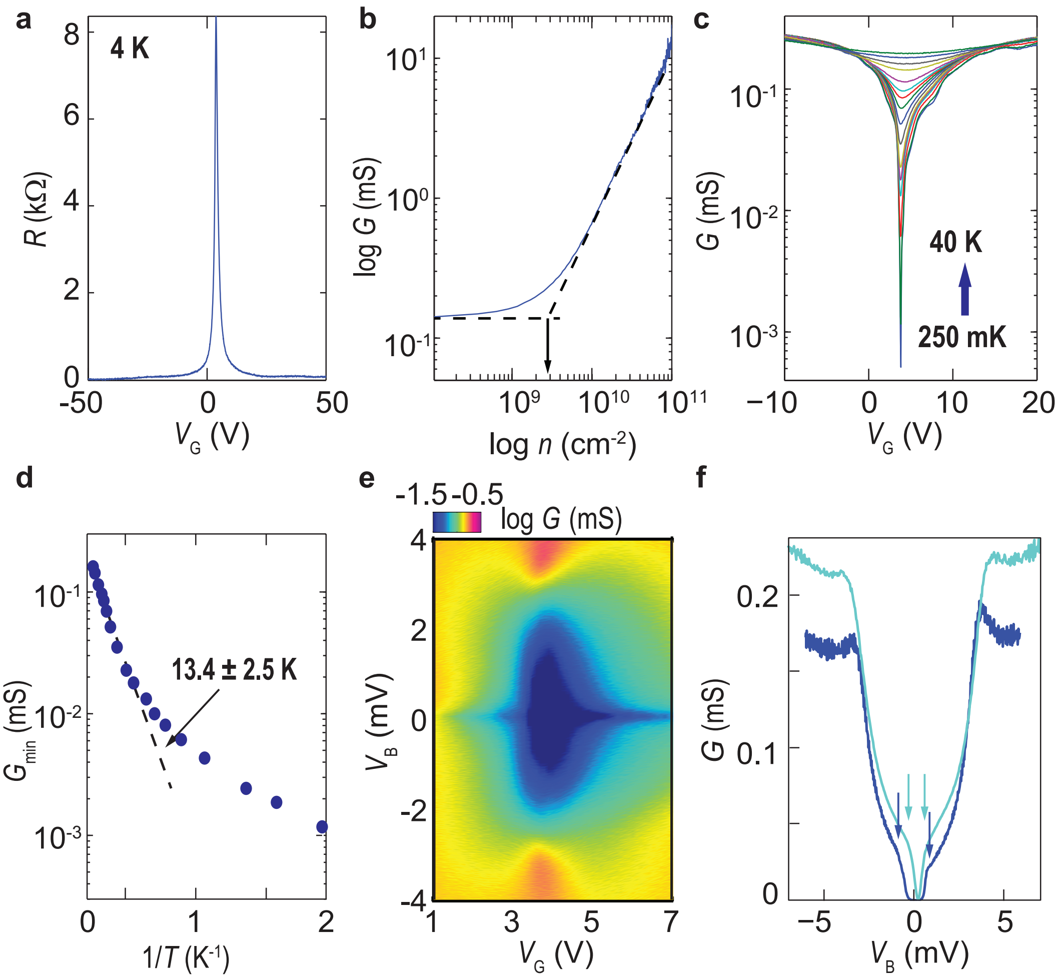

Supplementary Figure 1. **Insulating behaviour of multi-terminal suspended bilayer graphene. a,** Four-terminal resistance as a function of back gate voltage V_{G} measured at 4.2 K. **b,** Four-terminal conductance as a function of charge density n in a double logarithmic scale. The arrow points to the value of density of charge inhomogeneity, $n^* = 3 \times 10^9$ cm^{-2}. **c,** Temperature dependence of the conductance as a function of V_{G} measured in a two terminal configuration. **d,** Minimal conductance $\log(G_{\mathrm{min}})$ as a function of $1/T$, showing thermally activated behaviour of G_{min} above 2 K, with an activation energy of approximately 13 K. **e,** Color-plot of $\log(dI/dV)$ as a function of V_{G} and V_{B}, measured in a two-terminal configuration. **f,** The blue line corresponds to $G(V_{\mathrm{B}})$ extracted from the data shown in panel **e** at $V_{\mathrm{G}} = 3.8$ V. The cyan line represents the same measurement done using a different pair of contacts. Note the difference in low-energy behaviour (i.e., below the energy pointed to by the arrows): in one case the dI/dV is fully suppressed in the other not. In 4LG, we found the complete suppression irrespective of the contact pair chosen for the measurements.

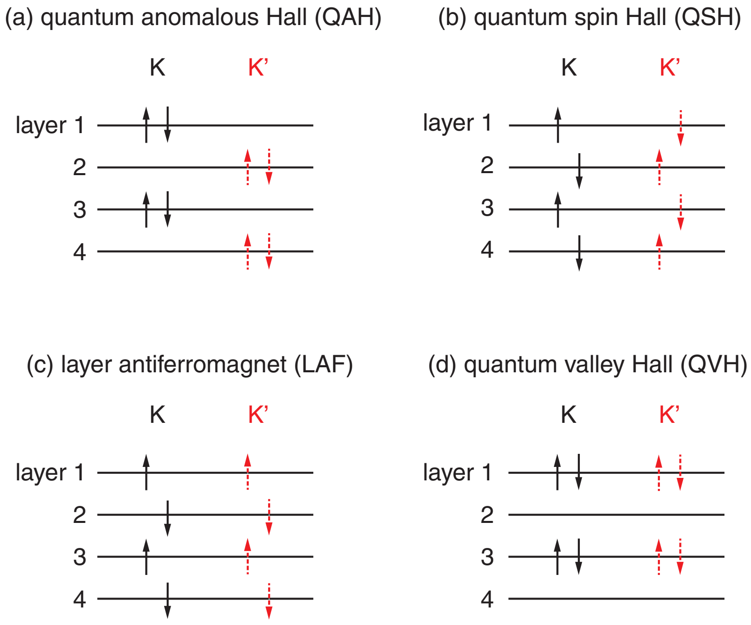

Supplementary Figure 2. **Broken symmetry states in tetralayer graphene.** Systematic representation of the spin and valley ordering of possible broken symmetry states in 4LG, based on the analogy with graphene bilayers

Supplementary Note 1

Insulating behaviour of suspended bilayer graphene

In the main text, we show that high-quality suspended 4LG becomes highly insulating at the CNP. We also state that the conductance suppression in 4LG is much more pronounced than in the insulating state of suspended bilayer graphene devices. Here, we substantiate this statement, by showing data obtained from a very high quality multiterminal suspended bilayer device, which can be directly compared to the data obtained from the 4LG devices discussed in the main text. Specifically, the device that we discuss here is one of the two exhibiting virtually identical behaviour, which have led to the first observation of the fractional quantum Hall effect in bilayer graphene (low-temperature carrier mobility ~1.000.000 $\mathrm{cm}^2 V^{-1} s^{-1}$ or higher; for details see Refs. 1 and 2). As for what regards the insulating state at $\mathbf{B} = 0$ T, the key features of our observations on bilayer graphene are in agreement with the behaviour reported earlier by others[3,4] (in those earlier studies, only two-terminal measurements have been presented).

Supplementary Fig. 1a shows the dependence of the four-terminal resistance on the gate voltage V_{G} measured at 4.2 K. At the CNP, the resistance is ~8 kΩ, much smaller than the value observed in 4LG (~300 kΩ). Note that, because of the smaller resistance, four-terminal measurements in bilayers are possible; two-terminal measurements have also been performed and exhibit – as far as the points discussed here are concerned – the same behaviour observed in four-terminal ones. The log-log plot of the four-terminal conductance as a function of charge density is shown in Supplementary Fig. 1b, and illustrates the extremely low density of charge inhomogeneity (as pointed to by the arrow, $n^* = 3 \times 10^9$ cm^{-2}). Supplementary Fig. 1c shows that the conductance G close to the CNP drops by three orders of magnitude as T is decreased down to 250 mK (i.e., the device is insulating); a logarithmic plot of the conductance at the CNP as a function of $1/T$ (Supplementary Fig. 1d) exhibits a thermally activated behaviour for T larger than a few Kelvin degrees. The activation energy – approximately 13 K – provides an estimate of the energy gap in the system, which is comparable to that found in 4LG (see the main text). The differential conductance (dI/dV) measured in a two terminal configuration as a function of V_{G} and bias voltage (V_{B}) is shown in Supplementary Fig. 1e. Similarly to 4LG, we find that the insulating state only occurs very close to the CNP, and that a small bias is sufficient to induce transport (independent of the configuration of the measurement, i.e. of which leads are used). Contrary to the case of 4LG, however, the detailed low-bias behaviour of the differential conductance in bilayers does depend somewhat on the specific pair of contacts used to perform the measurements (see two curves in Supplementary Fig. 1f). Irrespective of these differences, in all cases the low-bias suppression of the conductance in bilayers is not as pronounced as in 4LG, two energy scales can be distinguished (whereas it appears that in 4LG only one scale is visible in the low-bias data) and the transition from high to low differential resistance upon increasing V_{B} is less sharp.

Supplementary Note 2

AB-BA stacking faults as a possible origin of the insulating state in bilayer graphene

The insulating behaviour observed in high-quality suspended bilayer graphene is commonly considered to be a manifestation of electron-electron interactions that stabilize gapped, broken symmetry ground states. The current understanding of the phenomenon is incomplete, since the exact nature of the ground state is not known and is proving difficult to determine. Some additional uncertainty originates from the observation that in a number of experiments, high-quality suspended bilayers have been found to remain metallic at low temperatures.[5,6] Such metallic behaviour may be understood if strain is present in the bilayer, since, according to theory, strain may change the nature of the interaction-induced symmetry broken state. However, the observation of these metallic devices has led to the proposal of another scenario, according to which the insulating state observed in bilayer graphene is not a manifestation of electron-electron interactions, but a consequence of AB-BA stacking faults that are formed in suspended bilayers when the sample is cleaned by current-annealing[7] (AB-BA stacking faults are structural defects across which the stacking order of a bilayer changes from AB to BA). Since the low-energy electronic transmission across a stacking fault has been calculated to be very low, within this scenario, suspended bilayers remain highly conducting when no AB-BA stacking fault is formed, and become highly resistive when a stacking fault is present. As calculations show that the electronic transmission increases at larger energy, such a scenario predicts – at least qualitatively – an insulating temperature dependence of the conductance, with a resistance peaking around the charge neutrality. It may be argued that a similar mechanism may play a role in thicker graphene layers such as 4LG.

Here, we claim that attributing the observed insulating state to the presence of stacking faults is inconsistent with several aspects of the experimental observations made both on 4LG and on bilayer graphene, as well as with other observations already reported in the literature. The key arguments are:

1) The idea is that a stacking fault forms across the entire suspended graphene flake, so that the current necessarily has to cross it when the resistance of a device is measured in a two terminal configuration. In multi-terminal devices, however, we can measure the resistance between different pairs of contacts. It should be expected that only for some of the measurement configurations a stacking fault cuts across the current path. In other words, an insulating state should only be measured between the pairs of contacts separated by a stacking fault. Fig. 3b of the main text, however, shows that the device is uniform and that basically the same high resistance (i.e., the same insulating behaviour) is observed irrespective of the pair of contacts used to perform the measurements. In bilayers, we have also observed an insulating behaviour regardless of the measurement configuration.

2) The observed carrier density dependence of the resistance is not consistent with the energy dependence. In bilayers, as well as in 4LG, we find that the width – in carrier density – of the insulating state is approximately 2-3 $\times\, 10^{10}$ cm^{-2}, above which both bilayer graphene and 4LG become conducting. At such a density the Fermi energy is less than 100 μeV. In a single particle picture with a stacking fault, therefore, we should expect the differential conductance of the device to increase significantly when the bias is increased above 50-100 μeV. In contrast to this expectation, the bias required to increase the differential conductance is at least 10-20 times larger. Also the activation energy describing the temperature dependence is 10-20 times larger. This order of magnitude mismatch is inconsistent with the stacking fault scenario (the energy scales predicted by calculations are another order of magnitude larger).

3) Another indication comes from studies on suspended trilayer graphene devices, which have shown how the insulating state only appears in ABC-stacked trilayers, while Bernal-stacked trilayers always remain metallic.[8] If AB-BA stacking faults were created during current annealing, no experimental difference should be found when comparing ABC- and Bernal-stacked trilayers, because an AB-BA stacking fault converts one stacking order into the other.[9] Therefore, if stacking faults were the main cause of the insulating behaviour of Bernal-stacked trilayer should also exhibit insulating behaviour in a non-negligible number of cases, in contrast to the experimental observations.

Supplementary Note 3

Effect of staggered layer potential in multilayer graphenes

In this Section we briefly review the electronic properties of graphene multilayers that we have referred to in the main text, with a specific focus on the decomposition of the band structure into a collection of bilayer and monolayer-like bands. Most of the discussion is based on established results, which we include here for the convenience of the reader. However, the analysis of the effect of a staggered potential – which is a key issue here – is new. In particular, we show that the decomposition into bilayer and monolayer bands remains possible in the presence of a staggered potential and we analyze the implications of such a decomposition in this case.

The Hamiltonian of Bernal-stacked N-layer graphene can be decomposed into subsystems equivalent to mono- or bilayer graphene.[10–15] Specifically, the Hamiltonian of graphene with an odd number of layers N ($N = 2M + 1$) is decomposed into one monolayer-type and M bilayer-type Hamiltonians, and that of graphene with an even number of layers $N = 2M$ is decomposed into M bilayers without a monolayer-type subsystem. In the following, we present the explicit form of the decomposed Hamiltonian in the simplest approximation used in the main text, in which only the nearest intralayer coupling (γ_0) and the nearest interlayer coupling (γ_1) are taken into account. As shown below, this matrix decomposition is possible even in the presence of an interlayer staggered potential of the form $(-1)^j\Delta$ on the j-th layer. For non-zero Δ, all the effective bilayer bands open up energy gaps at the CNP, while the effective monolayer band remains ungapped. For even multilayers, the monolayer band is absent and, therefore, the spectrum becomes fully gapped in the presence of a staggered potential. This explains the behaviour observed in our study of 4LG, and previously in bilayers. The same argument explains why Bernal trilayer graphene – where a monolayer band is present – remains ungapped, and therefore conducting, at low temperature, as observed experimentally.

A unit cell of N-layer graphene is composed of A_j and B_j atoms on layer $j = 1, \cdots, N$. If the basis states are sorted as $|A_1\rangle, |B_1\rangle$; $|A_2\rangle, |B_2\rangle$; $\cdots$; $|A_N\rangle, |B_N\rangle$, the Hamiltonian for multilayer graphene reads:

$$\mathcal{H} = \begin{pmatrix} H_0 + U_1 & V & & & \\ V^\dagger & H_0 + U_2 & V^\dagger & & \\ & V & H_0 + U_3 & V & \\ & & \ddots & \ddots & \ddots \end{pmatrix}, \qquad (1)$$

with

$$H_0 = \begin{pmatrix} 0 & vp_- \\ vp_+ & 0 \end{pmatrix}, \quad V = \begin{pmatrix} 0 & 0 \\ \gamma_1 & 0 \end{pmatrix}, \quad U_j = (-1)^j \Delta \begin{pmatrix} 1 & 0 \\ 0 & 1 \end{pmatrix}, \qquad (2)$$

where v is the band velocity of monolayer graphene, $p_\pm = \xi p_x \pm ip_y$ with in-plane momentum (p_x, p_y) and the valley index $\xi = \pm 1$ ($+$ for K and $-$ for K'), and γ_1 is the nearest interlayer coupling. U_j describes the interlayer staggered potential, which vanishes in the absence of electron-electron interaction.

To decompose the Hamiltonian, we introduce a set of discrete wavenumbers along the layer-stacking direction,

$$\kappa_n = \frac{n\pi}{N+1}, \qquad n = 1, 2, \cdots, \left[\frac{N+1}{2}\right], \qquad (3)$$

where $[x]$ represents the largest integer which does not exceed x. We construct the wave basis as

$$|\phi_n^{(\mathrm{X,odd})}\rangle = C_n \sum_{j=\mathrm{odd}} (\sin \kappa_n j)|X_j\rangle\,,$$
$$|\phi_n^{(\mathrm{X,even})}\rangle = C_n \sum_{j=\mathrm{even}} (\sin \kappa_n j)|X_j\rangle\,, \qquad (4)$$

where $X = A$ or B, and the normalized factor $C_n = 1/\sqrt{2(N+1)}$ when $\kappa_n = \pi/2$, and $C_n = 1/\sqrt{N+1}$ otherwise. A superscript such as (A, odd) indicates that the wave function has its amplitude only on $|A_j\rangle$ sites in odd j layers.

By grouping the basis states as $\mathbf{u}_n = \{|\phi_n^{(\mathrm{A,odd})}\rangle, |\phi_n^{(\mathrm{B,odd})}\rangle, |\phi_n^{(\mathrm{A,even})}\rangle, |\phi_n^{(\mathrm{B,even})}\rangle\}$, the Hamiltonian Eq. (1) is block-diagonalized as $\mathbf{u}_{n'}^\dagger \mathcal{H}\mathbf{u}_n = \mathcal{H}_n\delta_{n'n}$, where

$$\mathcal{H}_n = \begin{pmatrix} \Delta & vp_- & 0 & 0 \\ vp_+ & \Delta & \tilde{\gamma_1} & 0 \\ 0 & \tilde{\gamma_1} & -\Delta & vp_- \\ 0 & 0 & vp_+ & -\Delta \end{pmatrix}, \tag{5}$$

and

$$\tilde{\gamma_1} = (2\cos\kappa_n)\gamma_1. \tag{6}$$

$\mathcal{H}_n$ is equivalent to the Hamiltonian of Bernal-stacked bilayer graphene with interlayer coupling $\tilde{\gamma_1}$. The staggered potential Δ enters here as a potential difference between the "upper" (the first and second) and "lower" (the third and fourth) layers of this effective bilayer. This is because the "upper" ("lower") wave bases are actually localized on the 1st, 3rd, 5th, $\cdots$ (2nd, 4th, 6th, $\cdots$) layers, so that a staggered potential gives effectively the same effect as the top-bottom potential asymmetry in bilayer graphene. The low-energy Hamiltonian is spanned by the basis states $\{|\phi_n^{(\mathrm{A,odd})}\rangle, |\phi_n^{(\mathrm{B,even})}\}$ (in analogy to $\{|A_1\rangle, |B_2\rangle\}$ for real bilayer graphene) and it is written as[16]

$$\mathcal{H}_n^{(\mathrm{low})} = -\frac{v^2}{\tilde{\gamma_1}}\begin{pmatrix} 0 & p_-^2 \\ p_+^2 & 0 \end{pmatrix} + \Delta\left(1 - \frac{2v^2p^2}{\tilde{\gamma_1}^2}\right)\begin{pmatrix} 1 & 0 \\ 0 & -1 \end{pmatrix}. \tag{7}$$

For $\Delta = 0$, this describes quadratic electron and hole bands touching at zero-energy, with effective mass $m^* = \tilde{\gamma_1}^2/(2v^2)$. The staggered potential Δ lifts the degeneracy at zero energy and opens an energy gap. For small momenta such that $2v^2p^2/\tilde{\gamma}^2 \ll 1$, the second term in Eq. (7) is approximately equal to $\Delta\sigma_z$, and Eq. (7) becomes equivalent to Eq. (1) in the main text.

The case of $\kappa_n = \pi/2$ is special in that the even bases $|\phi_n^{(\mathrm{X,even})}\rangle$ are identically zero and only two bases $\{|\phi_n^{(\mathrm{A,odd})}\rangle, |\phi_n^{(\mathrm{B,odd})}\rangle\}$ survive. The matrix for these two remaining bases is

$$\mathcal{H}_n = \begin{pmatrix} \Delta & vp_- \\ vp_+ & \Delta \end{pmatrix} \tag{8}$$

which is equivalent to the Hamiltonian of monolayer graphene. The staggered potential Δ does not open a gap at the band touching point but shifts the overall energy. This is because the wave bases are localized on the 1st, 3rd, 5th, $\cdots$ layers, and feel only $+\Delta$ part of the staggered potential.

For trilayer graphene ($N = 3$), for example, $\kappa_1 = \pi/4$ gives an effective bilayer and $\kappa_2 = \pi/2$ an effective monolayer as

$$\mathcal{H}_1 = \begin{pmatrix} \Delta & vp_- & 0 & 0 \\ vp_+ & \Delta & \sqrt{2}\gamma_1 & 0 \\ 0 & \sqrt{2}\gamma_1 & -\Delta & vp_- \\ 0 & 0 & vp_+ & -\Delta \end{pmatrix}, \quad \mathcal{H}_2 = \begin{pmatrix} \Delta & vp_- \\ vp_+ & \Delta \end{pmatrix}. \tag{9}$$

For tetralayer graphene ($N = 4$), $\kappa_1 = \pi/5$ and $\kappa_2 = 2\pi/5$ give a light-mass bilayer and a heavy-mass bilayer Hamiltonian, respectively, which read

$$\mathcal{H}_1 = \begin{pmatrix} \Delta & vp_- & 0 & 0 \\ vp_+ & \Delta & \lambda_1\gamma_1 & 0 \\ 0 & \lambda_1\gamma_1 & -\Delta & vp_- \\ 0 & 0 & vp_+ & -\Delta \end{pmatrix}, \quad \mathcal{H}_2 = \begin{pmatrix} \Delta & vp_- & 0 & 0 \\ vp_+ & \Delta & \lambda_2\gamma_1 & 0 \\ 0 & \lambda_2\gamma_1 & -\Delta & vp_- \\ 0 & 0 & vp_+ & -\Delta \end{pmatrix}, \tag{10}$$

where $\lambda_1 = (-1+\sqrt{5})/2$ and $\lambda_2 = (1+\sqrt{5})/2$. The energy band structures of trilayer and tetralayer graphene with and without Δ are illustrated in Figure. 5 in the main text. At low energy, the light-mass $\mathcal{H}_1$ and heavy-mass $\mathcal{H}_2$ bilayer Hamiltonians (10) may be written in basis states $\{|\phi_1^{(\mathrm{A,odd})}\rangle, |\phi_1^{(\mathrm{B,even})}\}$ and $\{|\phi_2^{(\mathrm{A,odd})}\rangle, |\phi_2^{(\mathrm{B,even})}\}$,

respectively, giving Hamiltonians of the form (7) with $\tilde{\gamma}_1 = \lambda_1\gamma_1$ and $\tilde{\gamma}_1 = \lambda_2\gamma_1$, and the staggered potential produces a $\Delta\sigma_z$ term for each bilayer.

According to our interpretation, the staggered potential responsible for the opening of the gap in tetralayer graphene (as well as in other even multilayers) originates from electron-electron interactions, which make the system at half filling unstable, in a way that is completely analogous to the case of bilayer graphene. A direct consequence of this idea is the even-odd effect that is seen experimentally, since –as we have shown here above– even multilayers open a gap close to the charge neutrality point, and odd multilayers remain ungapped. The analogy with the case of graphene bilayers that has been studied already in quite some detail[17–25] allow several broken-symmetry states to be suggested, as shown in Supplementary Fig. 2, depending on how the sign of the staggered potential changes upon switching spin and valley. In each panel, the spin and valley polarization of each layer is indicated by arrows with different directions and colors. The quantum anomalous Hall states (QAH), the quantum spin Hall states (QSH), and the quantum valley Hall (QVH) states have finite Hall, spin Hall, and valley Hall conductivity, respectively. The layer antiferromagnetic state (LAF) does not have any kind of Hall conductivity, but possesses a net magnetic moment on each layer. Among these candidates, the QVH is the only state which has a interlayer charge density modulation, and thus it is energetically unfavorable in terms of the cost of Hartree energy. The remaining three states, QAH, QSH and LAF, have a uniform charge distribution, and the ground state energy is lowered by exchange. In the continuum approximation, the exchange interaction works only between electrons with the same spin and valley,[24] so that the total energy is equal among those three states. Which of the three states is the actual ground state may depend on details, in complete analogy to what has been discussed for graphene bilayers

SUPPLEMENTARY REFERENCES:

---

1 D. K. Ki and A. F. Morpurgo, "High-quality multiterminal suspended graphene devices," Nano Lett. **13**, 5165–70 (2013).

2 D. K. Ki, V. I. Fal'ko, D. A. Abanin, and A. F. Morpurgo, "Observation of even denominator fractional quantum hall effect in suspended bilayer graphene," Nano Lett. **14**, 2135–9 (2014).

3 Jr. Velasco, L. Jing, W. Bao, Y. Lee, P. Kratz, V. Aji, M. Bockrath, C. N. Lau, C. Varma, R. Stillwell, D. Smirnov, Fan Zhang, J. Jung, and A. H. MacDonald, "Transport spectroscopy of symmetry-broken insulating states in bilayer graphene," Nat. Nanotechnol. **7**, 156–160 (2012).

4 F. Freitag, J. Trbovic, M. Weiss, and C. Schönenberger, "Spontaneously gapped ground state in suspended bilayer graphene," Phys. Rev. Lett. **108**, 076602 (2012).

5 A. S. Mayorov, D. C. Elias, M. Mucha-Kruczynski, R. V. Gorbachev, T. Tudorovskiy, A. Zhukov, S. V. Morozov, M. I. Katsnelson, V. I. Falko, A. K. Geim, and K. S. Novoselov, "Interaction-driven spectrum reconstruction in bilayer graphene," Science **333**, 860–863 (2011).

6 W. Bao, J. Velasco, F. Zhang, L. Jing, B. Standley, D. Smirnov, M. Bockrath, A. H. MacDonald, and C. N. Lau, "Evidence for a spontaneous gapped state in ultraclean bilayer graphene," Proceedings of the National Academy of Sciences **109**, 10802–10805 (2012).

7 P. San-Jose, R. V. Gorbachev, A. K. Geim, K. S. Novoselov, and F. Guinea, "Stacking boundaries and transport in bilayer graphene," Nano Lett. **14**, 2052–2057 (2014).

8 W. Bao, L. Jing, J. Velasco, Y. Lee, G. Liu, D. Tran, B. Standley, M. Aykol, S. B. Cronin, D. Smirnov, M. Koshino, E. McCann, M. Bockrath, and C. N. Lau, "Stacking-dependent band gap and quantum transport in trilayer graphene," Nat. Phys. **7**, 948–952 (2011).

9 M. Yankowitz, J. I. J. Wang, A. G. Birdwell, Y.-A. Chen, K. Watanabe, T. Taniguchi, P. Jacquod, P. San-Jose, P. Jarillo-Herrero, and B. J. LeRoy, "Electric field control of soliton motion and stacking in trilayer graphene," Nat. Mater. **13**, 786–789 (2014).

10 F. Guinea, A. Castro Neto, and N. Peres, "Electronic states and landau levels in graphene stacks," Phys. Rev. B **73**, 245426 (2006).

11 Sylvain Latil and Luc Henrard, "Charge carriers in few-layer graphene films," Phys. Rev. Lett. **97**, 036803 (2006).

[12] B. Partoens and F. Peeters, "Normal and dirac fermions in graphene multilayers: Tight-binding description of the electronic structure," Phys. Rev. B **75**, 193402 (2007).

[13] Mikito Koshino and Tsuneya Ando, "Orbital diamagnetism in multilayer graphenes: Systematic study with the effective mass approximation," Phys. Rev. B **76**, 085425 (2007).

[14] Hongki Min and A. H. MacDonald, "Chiral decomposition in the electronic structure of graphene multilayers," Phys. Rev. B **77**, 155416 (2008).

[15] Mikito Koshino and Edward McCann, "Parity and valley degeneracy in multilayer graphene," Phys. Rev. B **81**, 115315 (2010).

[16] E. McCann and V. I. Fal'ko, "Landau-level degeneracy and quantum hall effect in a graphite bilayer," Phys. Rev. Lett. **96**, 086805 (2006).

[17] H. Min, G. Borghi, M. Polini, and A. H. MacDonald, "Pseudospin magnetism in graphene," Phys. Rev. B **77**, 041407 (2008).

[18] O. Vafek, "Interacting fermions on the honeycomb bilayer: From weak to strong coupling," Phys. Rev. B **82**, 205106 (2010).

[19] R. Nandkishore and L. Levitov, "Dynamical screening and excitonic instability in bilayer graphene," Phys. Rev. Lett. **104**, 156803 (2010).

[20] R. Nandkishore and L. Levitov, "Quantum anomalous hall state in bilayer graphene," Phys. Rev. B **82**, 115124 (2010).

[21] R. Nandkishore and L. Levitov, "Flavor Symmetry and Competing Orders in Bilayer Graphene," ArXiv e-prints (2010), arXiv:1002.1966 [cond-mat.mes-hall].

[22] F. Zhang, H. Min, M. Polini, and A. H. MacDonald, "Spontaneous inversion symmetry breaking in graphene bilayers," Phys. Rev. B **81**, 041402 (2010).

[23] F. Zhang, J. Jung, G. A. Fiete, Q. Niu, and A. H. MacDonald, "Spontaneous quantum hall states in chirally stacked few-layer graphene systems," Phys. Rev. Lett. **106**, 156801 (2011).

[24] J. Jung, F. Zhang, and A. H. MacDonald, "Lattice theory of pseudospin ferromagnetism in bilayer graphene: Competing interaction-induced quantum hall states," Phys. Rev. B **83**, 115408 (2011).

[25] M. Kharitonov, "Antiferromagnetic state in bilayer graphene," Phys. Rev. B **86**, 195435 (2012).
